# In-patient comparison of marginal bone loss after 4 months in immediately loaded, submucosal, and transmucosal dental implants using 3D scanning: a prospective clinical trial

**DOI:** 10.1007/s00784-025-06255-y

**Published:** 2025-03-17

**Authors:** Armin Sokolowski, Martin Lorenzoni, Elisabeth Steyer, Felix Marschner, Anke Pichler, Alwin Sokolowski

**Affiliations:** 1 Division of Restorative Dentistry, Periodontology and Prosthodontics, Department of Dental Medicine and Oral Health, Medical University of Graz, Billrothgasse 4, Graz, 8010 Austria; 2 Department of Preventive Dentistry, Periodontology and Cariology, University Medical Center Göttingen, Göttingen, Germany

**Keywords:** Alveolar bone loss, Dental implantation, Endosseous, Imaging, Three-dimensional

## Abstract

**Objectives:**

This prospective observational study aimed to evaluate the clinical outcomes and impact of different treatment protocols - immediate loading using a round gold bar retained overdenture on two implants, submucosal healing, and transmucosal healing - on circumferential marginal bone levels around dental implants.

**Materials and methods:**

20 patients requiring six implants in the edentulous lower jaw were included. Within each patient, 2 implants were assigned to submucosal healing, transmucosal healing, or immediate loading protocols. Marginal bone loss (MBL) was evaluated using 3D scanning at implant placement and after 4 months while raising a full-thickness flap. Periotest values (PTVs), the oral health impact profile (OHIP-14) and regular clinical evaluations were conducted for up to 2 years.

**Results:**

The submucosal group exhibited the least mean vertical MBL (-0.22 mm), significantly lower than the immediate (-0.39 mm) and transmucosal (-0.36 mm) groups (*P* = .001). Peri-implant bone surface area reduction was lowest in the submucosal group (-0.87 mm²) compared to the transmucosal (-4.58 mm²) and immediate (-6.66 mm²) protocols (*P* = .002). One submucosal healed implant and two immediately loaded implants were lost. Immediate loading provided high patient comfort and demonstrated successful outcomes both in terms of clinical data (PTVs, MBL) and patient-reported outcome measures (OHIP-14, VAS).

**Conclusions:**

Submucosal and transmucosal healing protocols resulted in better marginal bone preservation compared to immediate loading. Although immediate loading enhanced patient comfort, it was associated with higher marginal bone loss.

**Clinical relevance:**

This study highlights the potential advantages of submucosal healing for marginal bone preservation and supports the use of three-dimensional scanning for precise bone loss evaluation, guiding clinical decision-making in implantology.

**Trial registration:**

ClinicalTrials.gov Identifier: NCT06408506.

## Introduction

The integration and stability of dental implants are paramount in reconstructive dental surgery, especially in the treatment of edentulous conditions. Traditional protocols, such as the Brånemark protocol, have emphasized the necessity of a stress-free healing period to ensure successful integration of implants. According to Brånemark and colleagues, this period is crucial to protect the implant from bacterial contamination and minimize mechanical loading during the initial healing phase, which typically involves a two-stage surgical procedure followed by a three to six-month healing period before exposure and functional loading of the implant [1–3].

Recent advancements and clinical outcomes have prompted a shift towards more immediate loading protocols, significantly reducing waiting time before the functional use of implants. This approach not only decreases the overall treatment duration but also enhances patient satisfaction by speeding up dental rehabilitation [4–7]. Immediate loading of implants, initially considered risky, has shown promising results comparable to those of traditional delayed loading, particularly in the anterior mandible where high success rates have been documented over the long term [8–10]. Emerging evidence suggests that immediate loading, when executed with precise clinical protocols such as the use of fixed superstructures or bar-retained overdentures to prevent non-axial loading, can achieve clinical outcomes similar to those of conventional methods [11]. The transgingival healing approach, well-documented and often applied for implants that demonstrate high primary stability but are not suitable for immediate loading, offers the advantage of eliminating an additional re-entry surgery, thus enhancing patient satisfaction [12].

Advanced implant surfaces that are moderately roughened from a grit-blasted titanium surface with additional chemical treatment, incorporating small amounts of fluoride ions into the titanium oxide layer, have shown favorable results and stable marginal bone levels over long-term study periods [13–15].

Maintaining adequate distances between dental implants is crucial for preventing negative influences. Insufficient implant-to-implant distances can impair blood supply to the surrounding bone, impacting the peri-implant bone regeneration which is essential for implant stability. Proper spacing ensures that each implant has enough room to integrate without adverse effects from neighboring structures, thus supporting better long-term success and stability of the implants [16].

Despite these advancements, there remains a lack of comprehensive data on the clinical outcomes of immediate loading with a round bar in edentulous mandibles. Specifically, it is unclear how different healing protocols - submucosal, transgingival, and immediate loading - affect marginal bone loss circumferentially around the implants. Existing studies typically use small-format radiographic measurements, which only provide mesial and distal point measurements, lacking the comprehensive three-dimensional assessment of bone loss [17, 18].

Current literature has not adequately addressed the precise, circumferential bone loss that occurs around implants under different healing protocols. Most studies rely on two-dimensional radiographs, which can lead to inaccuracies due to projection errors and fail to capture the complete peri-implant bone changes. Furthermore, there is limited information on the impact of using a round bar for immediate loading on two implants in the lower jaw, particularly in terms of its effects on bone resorption and clinical stability.

This study investigates the effects of three healing protocols - submucosal, immediate loading, and transmucosal - on marginal bone loss in edentulous mandibles within a prospective clinical trial. The primary focus is on changes in marginal bone levels from implant placement to the completion of osseointegration after four months, evaluated through three-dimensional circumferential digital measurements using three-dimensional scanning and computer-aided design (CAD) technology. Additionally, the study examines implant stability and patient-related outcomes (PROMs). A four-month period has been identified in the literature as optimal for achieving full osseointegration of the implants [19].

Understanding the full extent of marginal bone loss during the initial healing phase and the specific conditions under which it occurs is crucial for optimizing implant success and longevity. Addressing this gap could advance the field by providing evidence-based guidelines for selecting appropriate healing protocols, thus improving clinical outcomes and patient satisfaction.

The null hypothesis is, that there will be no significant difference in marginal bone loss among the three groups of implants with different healing protocols (submucosal, immediate, transmucosal).

## Materials and methods

### Trial design

This prospective study was conducted at the Department of Dental Medicine and Oral Health at the Medical University of Graz and included 20 patients requiring implant treatment for the edentulous lower jaw. Patients were selected based on predefined inclusion and exclusion criteria and were recruited from the regular patient pool seeking implant therapy at the institution, with no additional recruitment efforts.

### Participants

Eligible candidates required six implants in the edentulous mandible and presented at the outpatient clinic of the Department of Dental Medicine and Oral Health, Medical University of Graz. They underwent a comprehensive oral examination and their medical histories were reviewed to confirm compliance with the inclusion and exclusion criteria.

Inclusion criteria consisted of patients who were able and willing to provide written informed consent, were in good health as indicated by their medical histories, aged between 20 and 80, and possessed an edentulous mandible with adequate bone volume to support the placement of six implants and had a desire for implant-supported restorations. Sufficiently developed gingiva with at least 2 mm of keratinized mucosa in the area of the planned implants was required. Eligibility was further limited to individuals with complete mandibular dentures without any metal framework and bilateral balanced occlusion with stable occlusal contacts. In cases where the existing prostheses exhibited occlusal instabilities, adjustments were made prior to inclusion to ensure occlusal contacts on all teeth. The antagonist prosthetic treatment remained unchanged in all patients.

Exclusion criteria included homelessness, smoking, diabetes, medication with contraindications for implant therapy, periodontitis in the antagonist jaw, skeletal immaturity, any active malignancy or ongoing treatment for such, an active infection or the need for additional bone grafting at the operative site, persistent compartment syndrome or neurovascular residua of compartment syndrome, history of pathological fractures, contraindications to the class of devices under study such as known hypersensitivity, pregnancy, intention to become pregnant during the course of the study, breastfeeding, and lack of safe contraception. Patients receiving medication for anticoagulation purposes were also excluded to ensure optimal bleeding control during the intrasurgical assessments.

This trial was registered with ClinicalTrials.gov (NCT06408506). Approval of the study protocol was obtained in advance from the institutional review board (ethics committee, University of Graz; Graz, Austria; ref. 29-172ex16/17). The clinical procedures were conducted in conformity with the Helsinki Declaration and the ICH-GCP and STROBE/EQUATOR guidelines. Comprehensive information was provided to all participants about the planned surgical procedures and recall appointments, and all participants signed an informed consent form.

During the course of the study, 12 specific visits were defined as part of the standardized study protocol (Table 1). OHIP-14 was used to assess discomfort and oral health-related quality of life, while the visual analog scale (VAS) was employed to evaluate pain.

**Table 1:** Study visit schedule and assessments ^†^PT: Periotest values, ^‡^OHIP: oral health impact profile, ^§^VAS: visual analog scale

Visit	Timing	Description	Bone assessment	PT^†^	OHIP^‡^	VAS^§^	Probing
1	1 week preoperative	Preoperative assessment			X		
2	Implant placement	Implant placement	X	X			
3	2 days postoperative	Immediate loading				X	
4	1 week postoperative	Suture removal			X	X	
5	1 month postoperative	Follow-up			X	X	X
6	2 months postoperative	Follow-up			X	X	X
7	3 months postoperative	Follow-up			X	X	X
8	4 months postoperative	Implant exposure	X	X			X
9	1 week after re-entry	Suture removal				X	
10	6 months postoperative	Follow-up		X	X		X
11	1 year postoperative	Follow-up		X	X		X
12	2 years postoperative	Follow-up		X	X		X

Prior to surgery, each patient underwent a comprehensive preoperative evaluation, including medical history, clinical examination, and radiographic assessment using cone-beam computed tomography (CBCT) scans (Visit 1). This evaluation assessed the mandibular structure and bone quality, aiding in the selection of the appropriate implant diameters and lengths. During the initial visit, approximately one week before the scheduled implant surgery, patients received detailed information about the study’s procedures and examinations. Relevant demographic, medical, and dental data were collected. All implants were planned using Implant Studio (3Shape) to manufacture pilot surgical guides, ensuring safe distances from vital structures and at least a 3 mm marginal distance between implants. The implant positions were determined based on available bone volume and subsequent prosthetic treatment planning.

### Interventions

At Visit 2, all 6 implants were placed. All surgical procedures were conducted on an outpatient basis under local anesthesia and sterile conditions. Patients rinsed with 0.2% chlorhexidine digluconate (Chlorhexamed Forte; GlaxoSmithKline) preoperatively, followed by administration of local anesthesia using articaine with epinephrine 1:100,000 (Ultracain; Sanofi Aventis).

**Fig. 1:**
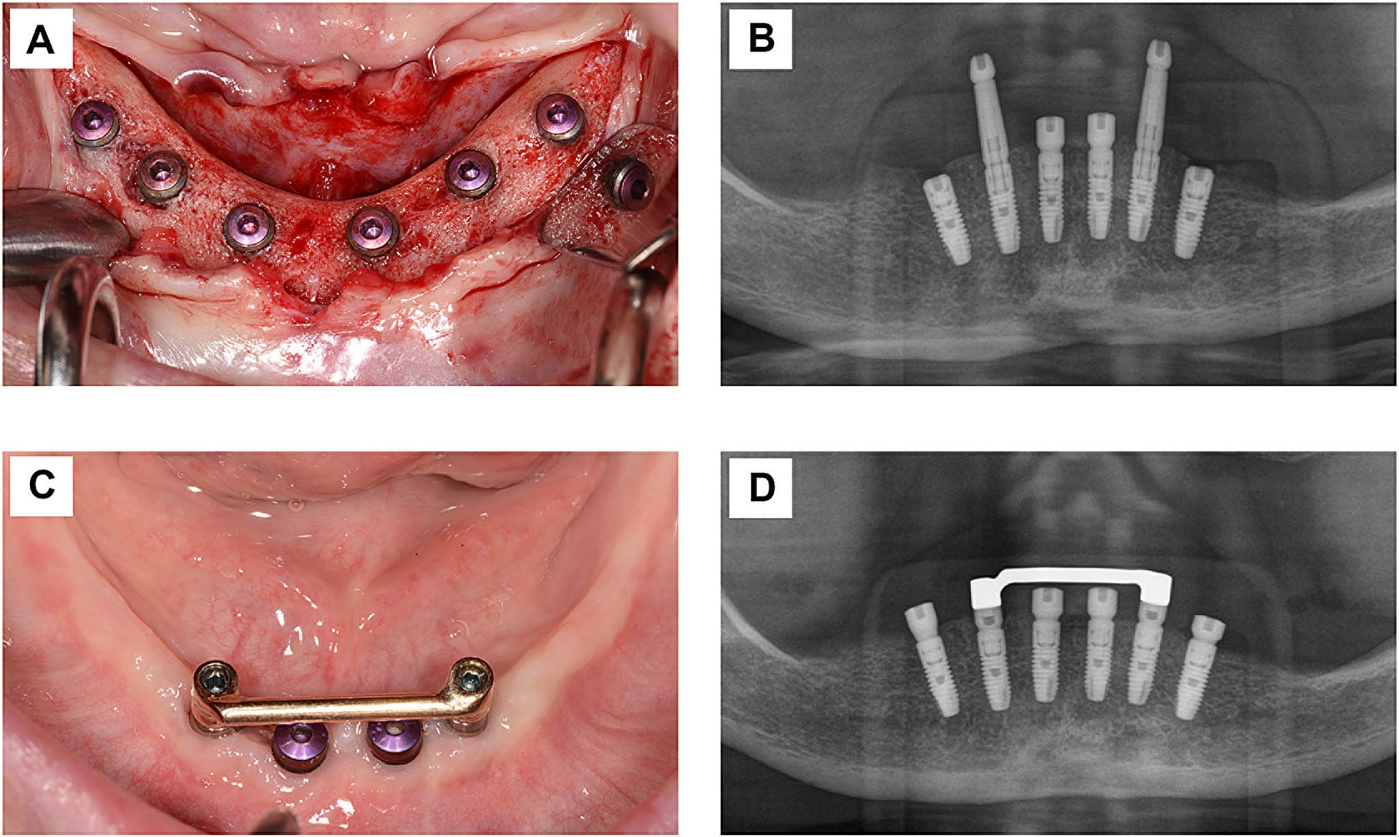
(**A**) Full-thickness flap after the re-entry procedure at Visit 8. (**B**) Radiograph showing implants with transfer abutments at positions 33 and 43, cover screws on distal implants, and healing abutments on mesial implants at Visit 2, following implant placement. (**C**) Clinical situation with integrated gold bar and healing abutments on transgingival implants at Visit 7. (**D**) Radiograph following re-entry surgery at Visit 8

A full-thickness flap was prepared to assess bone quality and quantity. Using the pilot guides, the implant positions were marked. CE-certified implants (ASTRA TECH Osseospeed EV, Dentsply Sirona Implants) were placed into six positions in the edentulous mandible, maintaining a minimum distance of 3 mm between each implant according to the manufacturer’s surgical protocol (Fig. 1A). The target torque for primary stability was between 35 Ncm and 45 Ncm. Four implants were placed interforaminally, and two additional implants were placed either interforaminally or more distally in patients with sufficient bone in the posterior region. The implant diameters used were either 3.6 or 4.2 mm, depending on the available bone volume. Implant stability was assessed using Periotest (Medizintechnik Gulden). To assess bone levels relative to the implant margin, intraoperative impressions were taken using sterile compound material. These impressions were later digitized for further analysis of circumferential peri-implant bone heights.

Cover screws were placed on the implants in the most distal positions for submucosal healing. The most mesial implants received healing abutments (Healing Uni EV, Dentsply Sirona Implants) with a height to emerge at least 1 mm for transmucosal healing, and ensuring no interference with the gold bars. The remaining two implants were initially fitted with transfer abutments for the impressions, followed by the placement of healing abutments until receiving immediate loading with round gold bars after a maximum of 72 h.

The conventional impressions were taken immediately after the implant placement using transfer abutments and Aquasil Ultra impression material (Dentsply Sirona) to manufacture the gold bars (Fig. 1B). A gold alloy cylinder (OD Cylinder EV, Dentsply Sirona Implants) and bridge screws were used by the laboratory technician to attach round gold bars (Friadent Gold Bar round, Dentsply Sirona Implants) using lasering welding (Fig. 1C). The completed bars were placed on index-free abutments (Uni Abutment EV, AstraTech Implant System EV, Dentsply Sirona Implants) and tightened to the recommended torque (25 Ncm) using the Uni Driver EV and Torque Wrench EV (Visit 3). The abutment height and design were identical in all patients. The associated prefabricated clips (Friadent Gold Bar Clip, Dentsply Sirona Implants) were directly polymerized into the patient’s complete dentures in their mouth, following the insertion of the bars and the use of block out wax (Periphery Wax, Sigma Dental).

Immediate postoperative care included the administration of oral antibiotics (Cefalexin or, if intolerant, Clindamycin) and a non-steroidal anti-inflammatory drug (NSAID) for pain relief (Dexibuprofen 400 mg, three times daily). Postoperative panoramic X-rays were taken to evaluate the surgical outcome. Sutures were removed approximately one week after surgery during follow-up visit 4. The healing phase lasted four months, accompanied by regular check-ups and clinical assessment of patient-related PROMs (Visits 5, 6 and 7).

Re-entry surgery was performed at visit 8, during which a full-thickness flap was raised to expose all implants and assess their integration and peri-implant bone contours, utilizing the impression technique from the initial surgery (Fig. 1A). Additionally, healing abutments were placed on the remaining submucosal healed implants to initiate the subsequent rehabilitation of all six implants. Prosthetic treatment was then performed on all six implants according to the patients’ preferences and predetermined therapy choices, which included fixed dental bridges on all six implants, removable individual bar-retained dentures, or Locator-fixed overdentures.

### Outcomes

PTVs were measured to assess implant stability immediately upon implant placement (visit 2), immediately after re-entry surgery at 4 months (visit 8), and at visits 10, 11, and 12, corresponding to 6 months, 1 year, and 2 years after implant placement. Radiographs were taken periodically to monitor the implants, including periapical radiographs in the right-angle technique. Additionally, panoramic radiographs were obtained after implant placement and four months later, following implant exposure (Fig. 1D). For patient-reported secondary outcomes, oral-health-related quality of life was assessed by having patients complete the OHIP-G14 questionnaire one week before implant placement and during recall appointments, while pain levels were evaluated using a visual analog scale (VAS).

Throughout the study, peri-implant tissues were palpated and visually inspected during patient visits to evaluate biological issues and peri-implant health, specifically looking for signs of inflammation such as redness and swelling. Gentle probing was performed to evaluate Bleeding on Probing (BOP) and pocket depths around all implants using a UNC15 probe (IMPLANT PROBE UNCPP15, Deppeler SA) made from polyether ether ketone (PEEK). Routine check-ups of the restorations were conducted to identify any technical issues, such as screw loosening, fractures, or similar complications.

**Fig. 2:**
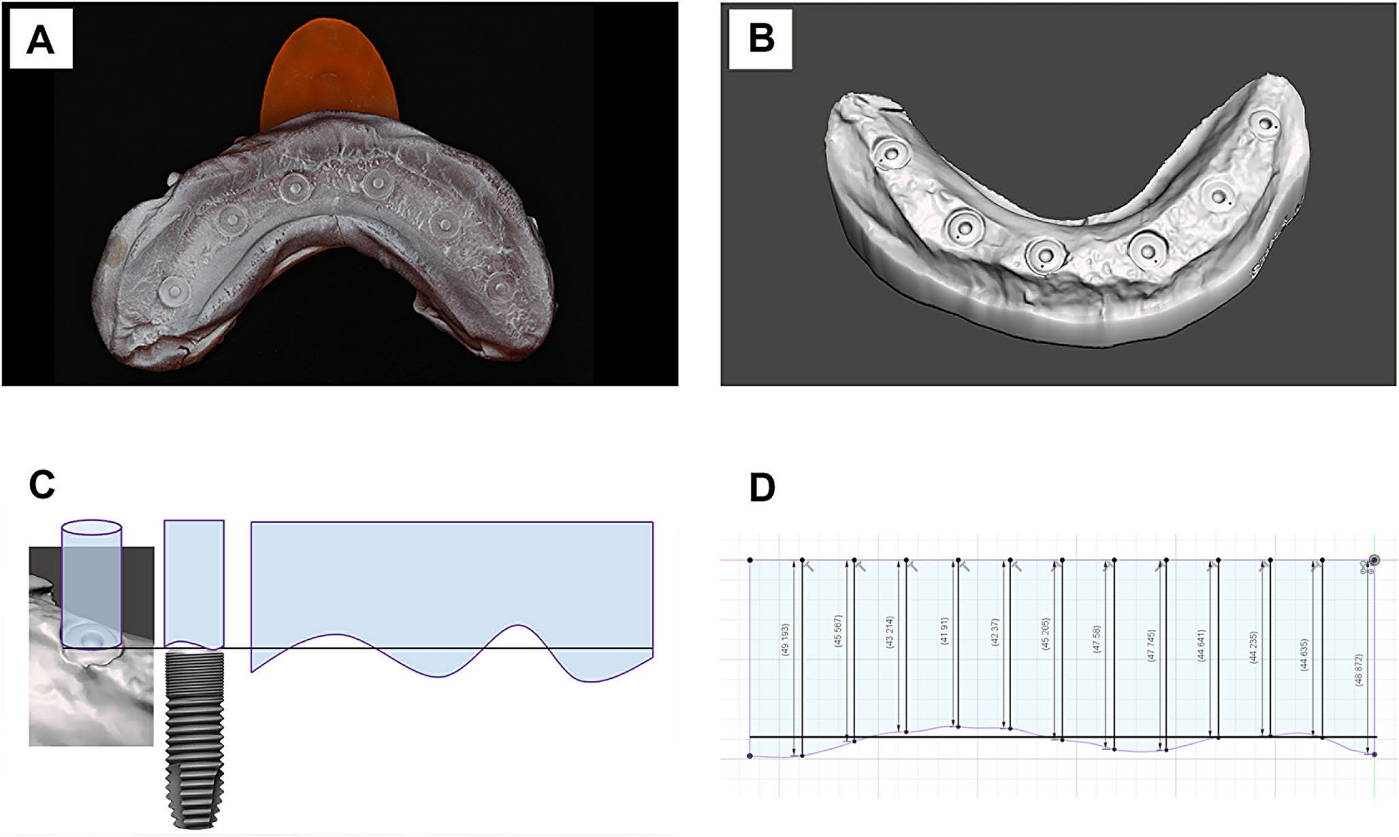
(**A**) Photograph of the impression compound coated with scanning powder. (**B**) Digital scan. (**C**) Schematic procedure for peri-implant bone measurements. (**D**) Exemplary bone contour around an implant cover screw level (black horizontal line) with 12 measurement points

The measurement technique employed a combination of intraoperative impression-taking and extraoral scanning to assess peri-implant bone conditions during implant placement and re-entry surgery (Visits 2 and 8). Initially, the bone environment was exposed using an open flap approach with buccal and lingual retention sutures. All implants were fitted with cover screws, and suction was used to control bleeding and keeping the peri-implant area clean. A thermoplastic impression compound (Kerr) was placed into a custom tray and heated to 55 °C in sterile saline to achieve the proper consistency for the impression. The tray was then left to cool for a few seconds before being applied to the surgical site. The compound was immediately cooled with room temperature sterile saline rinse after application to eliminate any residual risk of heat irritation. The impression was then removed, and later scanned using a high-precision lab scanner (D2000, 3Shape) after applying a thin homogeneous contrast layer of dental scan spray (CEREC Optispray Scan Spray, Dentsply Sirona) (Fig. 2A).

The digital scans were analyzed using Autodesk Fusion 360 software (Autodesk) to create a pattern of the peri-implant bone contour, delivering circumferential data on changes in the marginal bone level of the implants. (Fig. 2B) Fig. 2C illustrates the schematic procedure for peri-implant bone measurements. The computer-aided design (CAD) method involves constructing a cylinder around the peri-implant bone contour and unwrapping the cylinder to provide a planar representation of the circumferential bone profile [20]. Measurements were taken digitally at 12 positions around each implant, with measurement number 1 at the buccal side of each implant. Figure 2D shows an exemplary bone contour around an implant cover screw level (black horizontal line) with the 12 measurement points. On the right side of the jaw, the numbering proceeded in a clockwise direction, and on the left side in a counter-clockwise direction, so that measurement number 4 corresponded to the distal side, number 7 to the lingual side, and number 10 to the mesial side on both sides of the mandible.

Additionally, the overall area of bone loss around each implant was digitally recorded in square millimeters (mm²). It was considered that implants with a smaller diameter have a lower surface area, resulting in a mathematically smaller bone loss area compared to implants with larger diameters. This consideration was addressed by applying a correction factor to the calculated surface area (correction factor = 3.6/4.2 = 0.857 = 85.7%). Thus, in addition to the exact surface area, the results for implants with a larger diameter of 4.2 mm were multiplied by 85.7% for comparison.

This method, with its highly precise, objective, and standardized measurement process, allowed for the accurate assessment of marginal bone loss around the implants over the four months period and enabled a precise determination of whether significant differences existed among the three groups.

### Sample size

In a previous study at the same department, six months post-operatively, the median changes in bone levels showed a reduction of 0.91 mm in peri-implant bone height for loaded implants and 0.38 mm for non-loaded implants [21]. Based on this data, a sample size calculation was performed. A sample size of 8 in each group would have 80% power to detect a mean difference of -0.58 (the difference between a Group 1 mean, µ1, of -0.91 and a Group 2 mean, µ2, of -0.33), assuming a common standard deviation of 0.38, using a two-group t-test with a *P =. = .*05 two-sided significance level. Increasing the sample size in each of the 3 groups to 9 allows a one-way analysis of variance to achieve 80% power to detect a mean difference characterized by a variance of means of 0.06, assuming the common standard deviation is 0.38. Considering these statistical calculations, potential dropouts, and high demands for significant implications, a total patient sample size of 20 was determined.

### Statistical methods

All statistical analyses were carried out using statistical software (SPSS, version 29; IBM, Armonk, NY, USA) and spreadsheet software (Excel 2019, Microsoft, Redmond, WA, USA). Areas of lost bone were expressed as mean square millimeters and standard deviations (mm² ± SD). A Kolmogorov-Smirnov goodness-of-fit test was used to evaluate the samples for normal distribution. A *P*-value of *P* <.05 was defined as the cutoff for statistical significance. To compare the areas of bone remodeling and bone distances at 12 positions around the implants of the three groups, a one-way analysis of variance (ANOVA) was used. A general linear model with repeated measurements was applied to quantify the effect of visits on OHIP and VAS. The non-parametric Friedman test was performed to assess the change in PTVs over the visits.

## Results

### Baseline data

In total, 20 patients were included in the study, with an equal distribution of 50% female and 50% male. The mean age at the initial visit for males was 56.8 (± 11.8), with the youngest male patient being 32.3 years old and the oldest 75.9 years old. For females, the mean age was 63.2 (± 7.6), with the youngest female patient being 51.9 years old and the oldest 74.3 years old (Table 2).

**Table 2:** Patient demographics, ^†^N: number of patients, ^‡^%: percentage of total patients, ^§^SD: standard deviation

sex	*N* ^†^	%^‡^	Age at visit 1			
			Min	Max	Mean	SD^§^
male	10	50	32.3	75.9	56.8	11.8
female	10	50	51.9	74.3	63.2	7.6
total	20	100	32.3	75.9	60.0	10.2

In the antagonist jaw, 14 patients had complete dentures, 4 removable partial dentures, and 2 had fixed restorations.

After the re-entry surgery, 11 patients received bar-retained removable dentures on 6 implants, manufactured using selective laser melting (SLM). 3 patients received prostheses attached via Locator systems, and 2 patients received fixed bridges. In the remaining 4 patients, the gold bar was further adjusted to cover the additional implants, resulting in bar-retained dentures with gold bars and the same prostheses as before. In total, 75 implants with a diameter of 3.6 mm and 45 implants with a diameter of 4.2 mm were used. Table 3 shows the number of the implant diameters used as well as their percentage share for the 3 different groups. The group of immediately loaded implants had the highest proportion of implants with a smaller diameter, at 68%, although this was not statistically significant insertion torques for all placed implants ranged between 35 Ncm and 45 Ncm. Excessively high insertion torques were avoided by using accessory surgical drills in patients with dense bone, according to the manufacturer’s surgical protocol. Statistical testing showed no significant correlations between the diameter of the implant, the length of the implant, insertion torques, and the position of the implant.

**Table 3:** Implant distribution by diameter and healing protocol

Group	3.6 mm diameter		4.2 mm diameter	
Submucosal	22	55%	18	45%
Immediate	27	68%	13	33%
Transmucosal	26	65%	14	35%

### Outcomes

Throughout the two-year observation period of the study, a total of 3 implants were lost: 1 submucosal healed implant and 2 immediately loaded implants in one patient. Throughout the study, PTVs remained stable, and no technical complications occurred. BOP and deepest probing depth (DPD) did not differ significantly between the study groups (Table 4). Peri-implant soft tissue health remained stable in all patients, showing low to no bleeding on probing and no significant increases in DPDs. Slight loosening of the prostheses was resolved by lightly adjusting the bar clips. The patients maintained good oral health with the removable dentures and were consistently briefed during the healing period at monthly intervals (Visits 5, 6, and 7).

**Table 4:** Implant failures, Periotest values, bleeding on probing (BOP), and deepest probing depth (DPD) measured at various timepoints for each group, ^†^SD: standard deviation, ^‡^BOP: bleeding on probing, ^§^DPD: deepest probing depth

Implant failures	Group	*N*					
	Submucosal	1					
	Immediate	2	in one patient				
	Transmucosal	0					
Timepoints	Group	N	Mean Periotest	SD^†^ Periotest	BOP^‡^ (%)	Mean DPD^§^ (mm)	SD DPD (mm)
Implant placement	Submucosal	40	-7.7	0.65			
	Immediate	40	-7.7	0.61			
	Transmucosal	40	-7.7	0.83			
4 months after	Submucosal	39	-7.8	0.58	15	3.6	1.4
	Immediate	38	-7.5	0.76	11	3.3	1.2
	Transmucosal	40	-7.8	0.57	13	3.1	1.1
Half year	Submucosal	39	-7.5	0.73	8	3.0	1.1
	Immediate	38	-7.2	0.86	11	3.4	1.1
	Transmucosal	40	-7.6	0.55	10	3.1	1.3
1 year	Submucosal	39	-7.3	0.77	10	3.1	1.1
	Immediate	38	-7.5	0.56	13	3.3	1.2
	Transmucosal	40	-7.6	0.51	8	3.2	1.3
2 year	Submucosal	24	-7.3	0.98	9	3.5	1.3
	Immediate	24	-7.5	0.79	5	3.1	1.2
	Transmucosal	24	-7.5	0.68	14	3.2	0.9

The results detailed in Table 4 offer an in-depth perspective on the clinical observations of all 20 patients. During the entire follow-up period, the surrounding soft tissues remained healthy at all successfully integrated implant sites, with oral examinations consistently revealing no signs of peri-implant inflammation. Radiographic evaluations revealed that all integrated implants maintained stable marginal bone levels and exhibited favorable bone-to-implant contact.

**Fig. 3:**
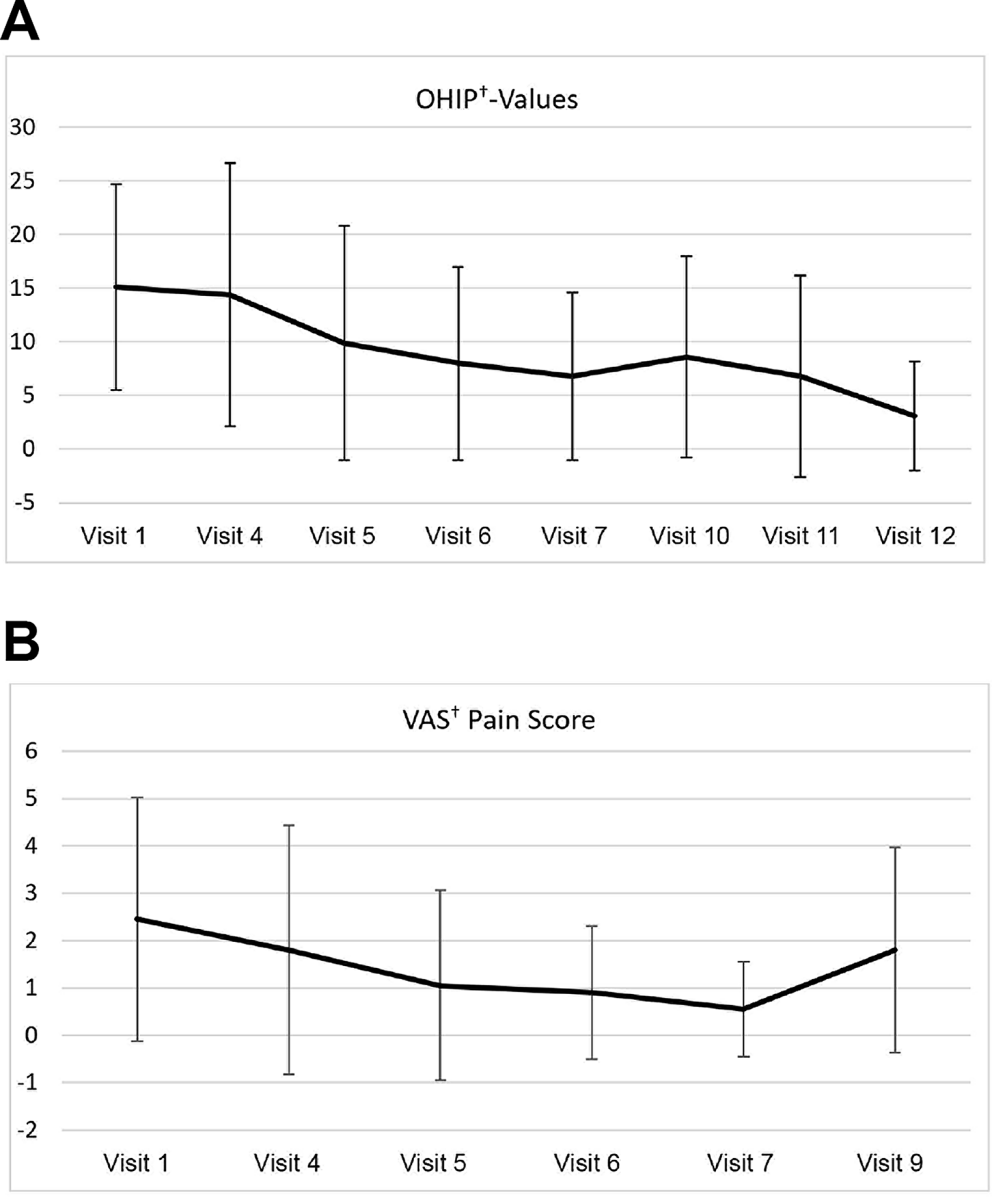
(**A**) Mean Oral Health Impact Profile (OHIP) scores at various visits. Error bars represent standard deviations. ^†^OHIP: Oral Health Impact Profile (**B**) Mean Visual Analogue Scale (VAS) pain scores at various visits. Error bars represent standard deviations. †VAS: Visual Analogue Scale

Patient-reported outcome measures showed decreasing values in OHIP and VAS pain scores. Patient-reported OHIP-G14 scores are illustrated in Fig. 3A, showing a decrease to 3.1 by 24 months. The general linear model with repeated measures indicated a significant decrease in OHIP scores between Visit 1 and Visit 12 (*P* <.001), with no significant influence of gender or age.

Figure 3B illustrates the patient-reported VAS pain scores, which improved similarly to the OHIP scores. The highest mean VAS score was 1.8 at Visit 3. VAS scores decreased during the course of the study but increased slightly one week after the re-entry surgery (Visit 9). The general linear model with repeated measures indicated a significant decrease in VAS scores between Visit 3 and Visit 9 (*P = .*003), with no significant influence of gender or age.

**Fig. 4:**
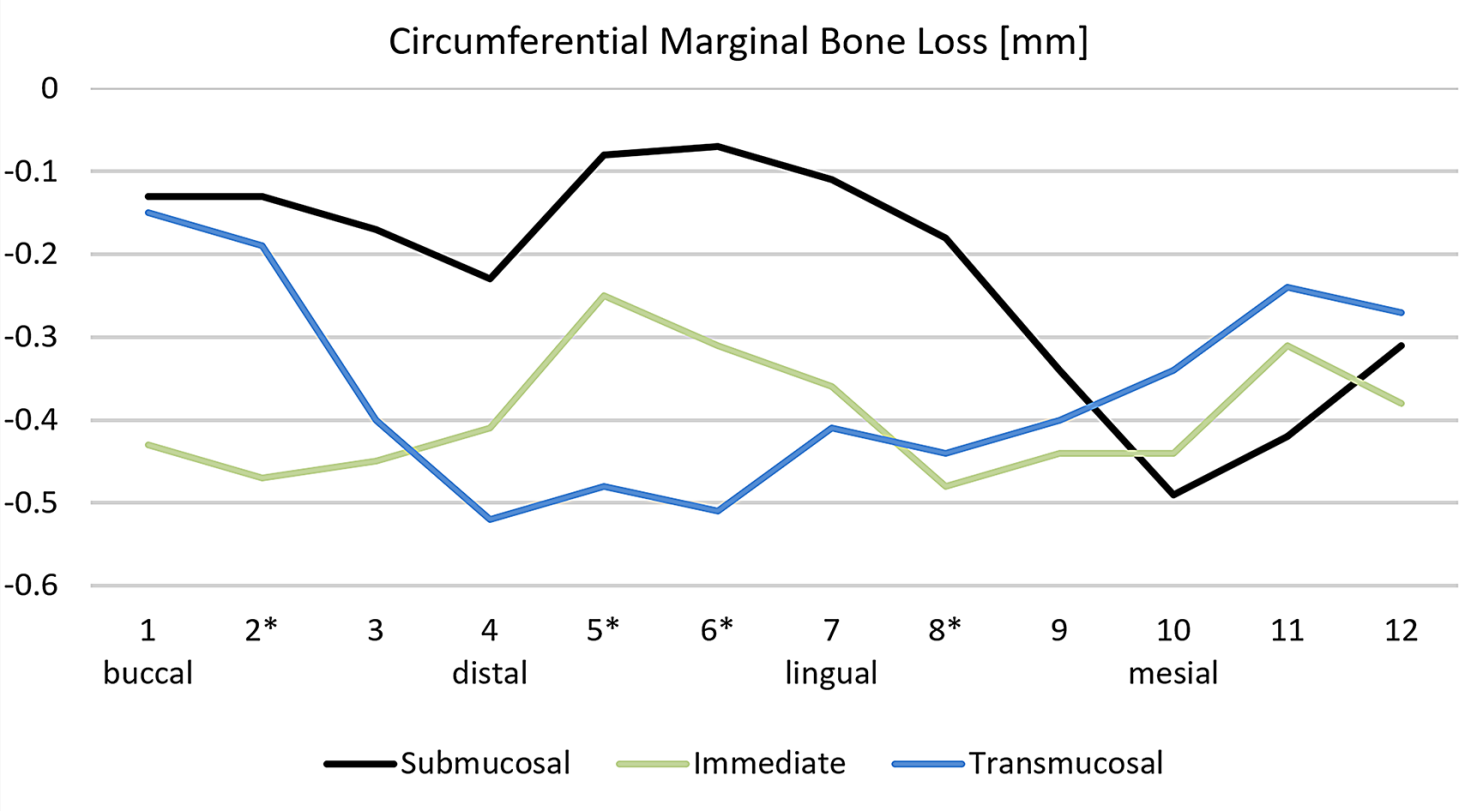
Mean vertical crestal bone loss in mm at various positions around the implants, *P <.05 indicates a statistically significant difference

Table 5 shows the digitally measured marginal bone loss circumferentially around the implants between the time of implant placement (Visit 1) and the time of re-entry surgery 4 months later (Visit 8). Figure 4 shows the mean values of bone loss for the implants, categorized into the three groups: submucosal healing, immediate loading, and transmucosal healing. It was observed that marginal bone loss was the least in the submucosal group at 9 out of 12 measurement sites. The differences were statistically significant at positions 2 (*P* = .039) and 8 (*P* = .017) compared to the immediate group, and at positions 5 (*P* = .027), 6 (*P* = .013), and 8 (*P* = .017) compared to the transmucosal group, with the submucosal implants showing the least bone loss in each case. The mean values of all 12 vertical measurements combined showed a statistically significant lower bone loss in the submucosal group of -0.22 mm compared to the immediate group of -0.39 mm and the transmucosal group of -0.36 mm (*P* = .001).

**Table 5:** Mean vertical crestal bone loss at various positions around the implants, ^†^: not significant, ^‡^: submucosal, ^§^: immediate, ^¶^: transmucosal

	Submucosal (mm)		Immediate (mm)		Transmucosal (mm)		*P*
Position	Mean	SD	Mean	SD	Mean	SD	
1 / buccal	-0.13	0.69	-0.43	0.57	-0.15	0.74	n.s.^†^
2	-0.13	0.57	-0.47	0.58	-0.19	0.72	*P* = .039 sub^‡^ vs. imm^§^
3	-0.17	0.53	-0.45	0.53	-0.4	0.6	n.s.
4 / distal	-0.23	0.62	-0.41	0.57	-0.52	0.55	n.s.
5	-0.08	0.67	-0.25	0.67	-0.48	0.66	*P* = .027 sub vs. trans^¶^
6	-0.07	0.55	-0.31	0.69	-0.51	0.68	*P* = .013 sub vs. trans
7 / lingual	-0.11	0.52	-0.36	0.61	-0.41	0.66	n.s.
8	-0.18	0.46	-0.48	0.52	-0.44	0.51	*P* = .017 sub vs. trans & imm
9	-0.34	0.51	-0.44	0.54	-0.4	0.54	n.s.
10 / mesial	-0.49	0.57	-0.44	0.59	-0.34	0.58	n.s.
11	-0.42	0.64	-0.31	0.56	-0.24	0.71	n.s.
12	-0.31	0.67	-0.38	0.6	-0.27	0.89	n.s.
all positions	-0.22	0.60	-0.39	0.58	-0.36	0.67	*P* = .001 sub vs. trans & imm

**Fig. 5:**
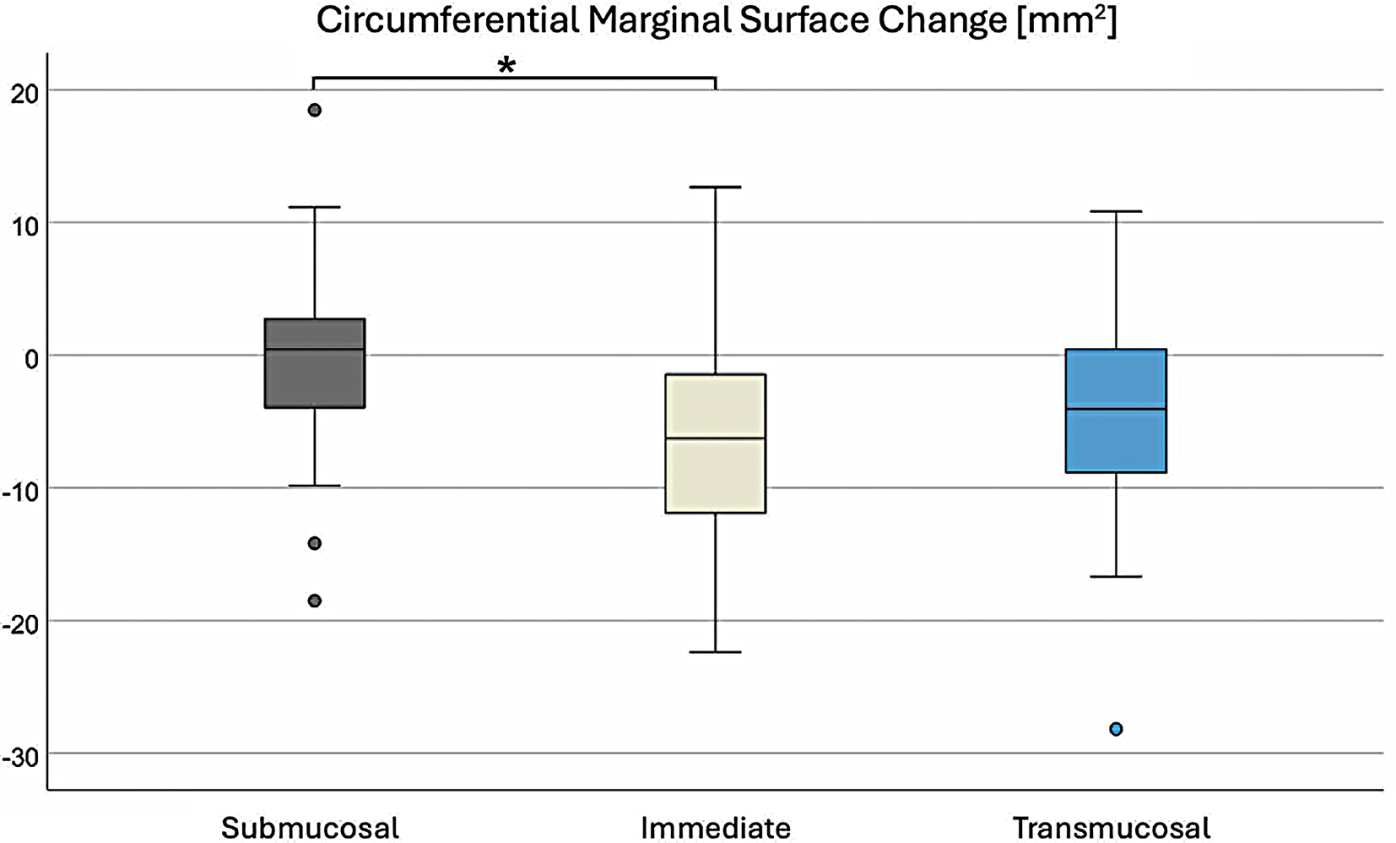
Circumferential marginal surface change in mm² around the three groups of implants with different healing protocols. *P <.05 indicates a statistically significant difference

Based on the circumferential digital measurement of marginal bone loss around the implants with different healing protocols, the bone loss could also be calculated as surface area. It was found that, regardless of the application of the correction factor described in the Methods section (surface area of marginal bone loss for implants with a diameter of 4.2 mm reduced to 85.7%), the submucosal group had the least reduction in peri-implant bone surface area, with − 1.08 mm² without the correction factor and − 0.87 mm² with the correction factor. In contrast, the surface area of the marginal bone loss was − 7.00 mm² without the correction factor and − 6.66 mm² with the correction factor in the immediate group, and − 4.96 mm² without the correction factor and − 4.58 mm² with the correction factor in the transmucosal group (Table 6). The one-way analysis of variance showed a significant difference between submucosal and immediate loading in both cases of exact surface measurement and with the application of the correction factor (*P* = .002). Figure 5 graphically presents the results of the bone loss surface areas as box plots using the correction factor.

**Table 6:** Mean peri-implant bone surface area reduction for submucosal, immediate, and transmucosal groups, with and without diameter correction

		no diameter correction		diameter correction	
	N	mean bone loss (mm²)	SD (mm²)	mean bone loss (mm²)	SD (mm²)
Submucosal	39	-1.08	7.07	-0.87	6.65
Immediate	38	-7.00	7.87	-6.66	7.53
Transmucosal	40	-4.96	8.01	-4.58	7.31

## Discussion

The primary objective of this study was to prospectively evaluate the impact of three different healing protocols - immediately loaded, submucosal, and transmucosal - on marginal bone levels around dental implants, and to assess the clinical outcomes of immediate loading using a gold bar. This bar, round in cross-section, was designed to allow tilting of the prosthesis, mitigating forces on the implants to protect osseointegration. Immediate loading of implants has attracted attention for its potential to shorten treatment duration and enhance patient comfort by eliminating the need for a second surgical procedure. In alignment with findings from various clinical studies, immediate loading can achieve success rates comparable to conventional two-stage protocols, provided that adequate primary stability is attained [7, 22–24].

However, some authors, such as Weber et al. note that immediate loading may introduce higher initial stress on the bone, which could contribute to early marginal bone loss [25]. The results of the present study reflect this tendency, as the immediately loaded implants showed slightly higher marginal bone resorption compared to the submucosal and transmucosal approaches. The submucosal healing approach, in which the implant is completely covered by the mucosa during healing, resulted in the lowest marginal bone resorption after four months. A similar protective effect has been reported in other studies on submucosal healing protocols, where the absence of early exposure to the oral environment appears to minimize initial bone remodeling [26, 27].

Nonetheless, this early advantage may decrease once the implant is uncovered, as the subsequent surgical procedure and prosthetic loading can trigger additional bone remodeling. The transmucosal healing approach, whereby the implant penetrates through the mucosa but remains unloaded, also demonstrated successful osseointegration with relatively minimal bone loss. These observations concur with previous research indicating that transmucosal protocols can be a reliable one-stage alternative, reducing surgical interventions while protecting the implant from masticatory forces [28].

In clinical practice, the choice of the healing protocol depends on a range of factors, including bone density, soft-tissue conditions, and patient preferences. This underscores the need for individualized treatment planning. Primary stability and insertion torque are critical for successful immediate loading, however, no universal minimum torque threshold has been definitively established in the literature. Schnitman et al. emphasized the importance of primary stability, particularly in regions such as the anterior mandible, where bone density is typically higher [29].

Whereas several studies favor insertion torque values between 30 and 35 Ncm [30, 31], others accept broader ranges, such as 20–80 Ncm, depending on clinical and anatomical conditions [32–35]. These values and the required primary stability are influenced by several factors, including bone density, implant shape and design, surface characteristics, and the surgical technique employed [36].

However, excessively high torque may cause compression of the bone, leading to reduced blood perfusion and increased bone remodeling [37].

The protocol applied in the study, using a gold bar, may enhance primary stability by distributing occlusal forces across both implants. However, the failure of one implant can negatively affect the stability of the remaining implant, as was the case with the simultaneous loss of two implants in this study.

To enhance the reliability of the study outcomes, a precise measurement technique was used to minimize investigator-dependent biases [20]. Intraoperative impressions were obtained and analyzed using a digital protocol by an investigator blinded to clinical details. This approach enabled three-dimensional assessment of peri-implant bone via digital surface impressions, providing objective, circumferential measurements and reducing errors from radiographic or operator bias. Circumferential digital measurement of marginal bone loss around the implants allowed for comprehensive analysis and offered new insights into the characteristics of bone loss. Since it requires an open-flap procedure, this method is generally limited to situations where second-stage surgery is already indicated.

In the immediate placement of implants following tooth extraction, a retrospective study by Lee et al. compared the effects of different loading protocols on marginal bone changes over a 24-month follow-up period [38]. Marginal bone levels were assessed using periapical radiographic images, with measurements taken at direct bone-implant contact points and distant crestal points. The results demonstrated that immediately loaded implants exhibited more marginal bone loss at the bone-implant contact points compared to conventionally loaded implants. These findings align with the results of the present study, further supporting the influence of loading time on peri-implant bone stability.

Each healing method displays indications based on the clinical scenario, and the use of torque-controlled surgical equipment allows for a predictable and precise surgical workflow. The findings of the present study are clinically relevant as they highlight the need to balance the benefits of reduced marginal bone loss with the advantages of immediate loading, such as faster rehabilitation and improved patient comfort. Although the observed difference of approximately 0.2 mm in marginal bone loss between protocols appears small, it could become significant over the long term, potentially leading to thread exposure, higher risk of peri-implant disease, and cosmetic concerns. On the other hand, immediate loading protocols offer considerable advantages in terms of patient satisfaction, particularly when rapid restoration and retention of provisional prostheses are a priority [39].

There are some limitations in this study that need to be addressed: While the re-entry surgery was performed four months after implant placement, a longer follow-up might yield more comprehensive data on long-term bone stability. However, this would require either postponing the re-entry or adding a third open-flap procedure solely for research purposes, raising ethical concerns due to the invasive nature of the intervention. The bone assessment technique was necessarily invasive, since it required an open-flap procedure, and it depended on the operative site and patient compliance. Although peri-implant bone remodeling can be minimal (0.1–0.5 mm), factors such as bleeding and residual soft tissue may have introduced slight variations. In order to minimize inaccuracies, advanced CAD-software was used. Although standardizing implant dimensions might have provided more consistent results, it could have compromised patient-specific treatment decisions. Variables such as general health, medication, keratinized mucosa, abutment height and design, and inter-implant distance were carefully controlled to maintain homogeneity and reduce bias. The prosthetic delivery using round gold bars, though efficient and quick, is equipment- and technique-sensitive. The impressions need to be precise, and the technician must be skilled in laser-welding gold copings. Prefabricated parts allow immediate loading within 72 h, but delays can expose implants to rotational forces during re-screwing, affecting stability and remodeling. Milled bars, although more technique-sensitive and time-consuming, offer a better fit, improved prosthesis retention, and enhanced cleanability compared with prefabricated bars [40]. However, providing a high-quality milled bar within 72 h remains challenging. Despite these constraints, the findings offer valuable insights into marginal bone dynamics and immediate-loading protocols.

Future studies could refine these digital methods or adopt less invasive imaging for longitudinal peri-implant assessment. The precise circumferential measurement technique performed in this study might also advance research on implant design, surface characteristics, and surgical protocols.

## Conclusions

This study demonstrates that the choice of the healing protocol significantly affects marginal bone levels around dental implants. Submucosal healing resulted in the least marginal bone loss, while immediate loading, although clinically successful, showed higher peri-implant marginal bone resorption. The use of precise digital measurement techniques provided objective and comprehensive data, underscoring the need for tailored implant therapy based on individual clinical scenarios. Prospective research should focus on long-term outcomes and standardized methodologies to further refine these protocols and improve patient care in dental implantology.

## Data Availability

No datasets were generated or analysed during the current study.
